# Red Rubber Stomatitis

**Published:** 1913-04-15

**Authors:** J. W. Dent


### RED RUBBER STOMATITIS.

BY J. W. DENT, L.D.S.I.

During the spring of last year, a married lady about 40 to 45 years of age consulted me about the state of her mouth, which was very much inflamed. She was wearing a complete set of teeth in vulcanite, probably Ash's brown rubber, with a pink, which might be the Company's gum pink. There was nothing remarkable about the plates either as to color or weight or fit, and nothing to suggest that they were underbaked or in any way likely to cause the condition her mouth presented, and after advising a mouth-wash I asked her to call again. A few weeks later I saw her and she was suffering from sore throat, a swollen tongue, and stomach trouble, with pain and sickness, and was in the doctor's hands, feeling very sorry for herself. After that, she changed her medical adviser several times, and amongst others consulted a high authority in Newcastle; he, with at least one other, told her the plates were too heavy and should be made lighter. Of course I was skeptical, but said it was just possible that the red rubber might have something to do with it, and offered to make them in pure black rubber. I missed her for a long time, during which she had been to Sir Henry Butlin, who at once said, "I have seen several cases of this kind. You are being poisoned by those red plates and should get some black ones" (or words to that effect.) (She had worn the plates for twelve years and had a sore tongue for several years, but put off coming to me.)

In October, I made her upper and lower plates in pure black rubber. She left town rather unexpectedly after that, so that I did not see her again, but in March I wrote to her and, in reply, she said that from the time the plates were changed her mouth and tongue healed and her general health improved. She also said that during November for a month or so after the plates were changed, her tongue seemed to lose all sensibility and she had no sense of taste. However, when she wrote me in March, she was in good health, and very thankful to be out of the misery.

There seems to have been very little definite local indication of mercury poisoning. She complained mostly of the swollen tongue and throat and of the stomach troubles. There had never been any sore or ulcer, only redness and swelling, and I could get no evidence of salivation. Except for the high authority from which the diagnosis came, I should not have troubled you with this case, but considering that factor and the prompt disappearance of the symptoms, I thought it might be well to hear if others had encountered similar cases. Should Sir Henry have been right, then we may have been missing opportunities of giving relief.

He prescribed a paint to be applied two or three times a day composed of:

| | |
|---|---|
| Menthol | mv |
| Ol. pini sylvestris | mv |
| Ol. olive | ʒii |
| Christy's benzoinol | ad. ℥i |

and advised her to squeeze a little 'Dartring' toilet lanoline into her mouth occasionally.

